# Influence of strata-specific forest structural features on the regeneration of the evergreen broad-leaved forest in Tianmu Mountain

**DOI:** 10.1371/journal.pone.0247339

**Published:** 2021-02-23

**Authors:** Junsong Long, Mengping Tang, Guangsheng Chen

**Affiliations:** 1 College of Environmental and Resource Sciences, Zhejiang A&F University, Hangzhou, Zhejiang, China; 2 Key Laboratory of Carbon Cycling in Forest Ecosystems and Carbon Sequestration of Zhejiang Province, Zhejiang A&F University, Hangzhou, Zhejiang, China; Tennessee State University, UNITED STATES

## Abstract

The vertical stratification of the stand may lead to a high heterogeneity of microenvironment in the forest, which further influences the understory regeneration and succession of the forest. Most relevant previous studies emphasized the overall effects of the Whole-stand structural characteristics on understory regeneration, while the strata-specific impacts of the overstory should be explored especially for those forests with a complicated combination of overstory species and heights. In this study, a subtropical evergreen broad-leaved forest in Tianmu Mountain of China was intensively investigated within 25 plots of 20 m × 20 m, aiming to find out how significant the stratified overstory (trees with diameter at breast height (DBH) ≥ 5 cm) structure and non-structure characteristics impact the understory (trees with DBH < 5 cm) regeneration. Regardless of species composition, the studied overstory was evenly divided into three strata (*i*.*e*. upper, middle and lower strata) according to their heights. Redundancy analysis was applied to explore both overall and strata-specific forest structure on characteristics (height, DBH, species diversity, and density) of tree regeneration. We found that the overall effect of the whole overstory on the forest regeneration depended mostly on diameter at breast height (DBH), tree species richness index and crown width. However, when analyzing with the strata-specific characteristics, the most pronounced impact factors for the regeneration were tree height of the upper and lower forest strata, tree species richness index and crown width of the middle and lower forest strata, and the competition index impact of the lower forest stratum. Among the three strata, the lower forest stratum showed the most significant impact with three characteristics on the understory regeneration, which may be attributed to their direct competition within the overlapping near-ground niches. Among the new generations, seedlings and saplings were more sensitive to the overstory structural characteristics than young trees. Our results suggest that the overstory showed strata-specific effects on the understory regeneration of evergreen broad-leaved forests in subtropical China, which provides theoretical basis for strata-specific forest management in similar forests.

## Introduction

The evergreen broad-leaved forest is one of the primary forest types in China. Due to irrational planning and utilization of forest resources, the area of the evergreen broad-leaved forests has been continuously decreasing [1]. The transformation of the evergreen broad-leaved forests into other forests or degradation causes many ecological problems, e.g., loss of ecological functions [2]. Natural regeneration is the most applied method to regenerate the evergreen broad-leaved forests in China [3]. Forest natural regeneration undergoes several developmental stages, e.g., seed production and dispersal, seed germination, seedling establishment and growth [4, 5]. Each stage will be influenced by many impact factors, especially light, litter depth, soil and stand conditions. Moreover, seedling establishment and growth largely determine the current situation and succession direction of the stand, so the research on the impact factors of seedling establishment and growth has attracted much attention.

The stand structure is one of the main driving factors for tree regeneration [6, 7], which plays a key role in the succession and recovery of the forest [8]. The stand structure includes a spatial and non-spatial structure. Due to the simplicity of non-spatial structure calculation, most studies used the non-spatial structure variables. Non-spatial structure variables can describe the average stand characteristics, which were not affected by the relative position of neighbouring trees [9]. The previous studies indicated that the smallest beech seedlings regeneration was determined by stand structure variables to a greater extent [10], and the increase of seedling height and dry mass was greater in the sparse shelterwood than in the dense shelterwood [11]. Chen and Cao [12] indicated that stand density had no significant effect on the number, base diameter, and height of *Pinus tabulaeformis* seedlings. Large tree basal area of forest was not conducive to regeneration [13, 14]. These previous studies mainly assessed the relationship between horizontal non-spatial structure variables and regeneration [15].

Because the forest vertical structure largely determines the differences in the distribution of resources such as water, heat, light, and nutrients in the forest [16], it has an important effect on species growth, reproduction, death, and resource utilization [17–19]. Therefore, many researchers have begun to explore the impact of forest vertical structure on regeneration. For example, the higher the forest vertical structure diversity is, the more favourable regenerated trees are [20]. Tall trees in the forest were not suitable for regenerated tree development in uneven-aged northern hardwoods [13]. The moderately dense canopy may be beneficial for tree regeneration, rather than aggressive shade-intolerant graminoid or forbs [21]. In the old-growth lowland rainforest of Namdapha National Park in north-east India, the mid-canopy and low-canopy species were found to have higher survival rates than the top-canopy species [22]. The less proportion of small trees was less conducive to seeding regeneration [23]. Fir regeneration was more abundant in patches with a higher proportion of larger trees [24]. Ou et al. [14] considered that the crown index, large and small tree proportions had no significant effect on the number of *Excentrodendron hsienmu* seedlings, but the effect on the seedling diameter and tree height was significant. The overstory structure of the stand determines the change of microclimate in the stand, and structural elements have a stronger influence on microclimate conditions than tree species composition of the overstory [25].

The studies for forest spatial structure have particular significance in forest protection and management [26], which can provide a deeper understanding of tree patterns and determine the properties of the ecosystem [27]. With the gradual improvement of forest management level, stand spatial structure based on the relationship of neighbourhood trees is one of the research priorities. A few researchers have carried out the research on the relationship between spatial structure based on the relationship of neighbourhood trees and regeneration [28]. Experiments showed that the average DBH and tree height of *P*. *koraiensis* seedlings in all experiment sites increased with the increase of opening degree in the same aspect [29]; medium mingling and random distribution were suitable for artificial regeneration of *Pinus koraiensis* seedlings in the secondary forest [28]. Spatial pattern analysis showed that there was a positive spatial relationship between mature Lebanese cedar trees as well as between mature and juvenile cedars [30]. Through quantifying the competitive influence of neighboring trees, Saha et al. [31] indicated that the height to DBH ratio of young oak (*Quercus robur*) significantly increased with aggregate and intraspecific competition, but interspecific competition had no significant effect on the height to DBH ratio.

Great achievements have been made in studying the effects of spatial distribution and attributes of the whole stand on natural regeneration, but the impact of spatial distribution and attributes of each forest stratum on regeneration is still unclear. Understanding the impact of the spatial and non-spatial structure of each forest stratum on regeneration is crucial to formulate forest management measures that can encourage natural regeneration and succession process. Therefore, the main objectives of this study were to: (1) identify the effects of the most important structural factors on tree regeneration at forest stand and stratum levels, and (2) reveal the response patterns of regenerated trees to the leading forest strata structure indicators at different growth stages.

## Methods

### Study area and experimental site

**Fig 1:**
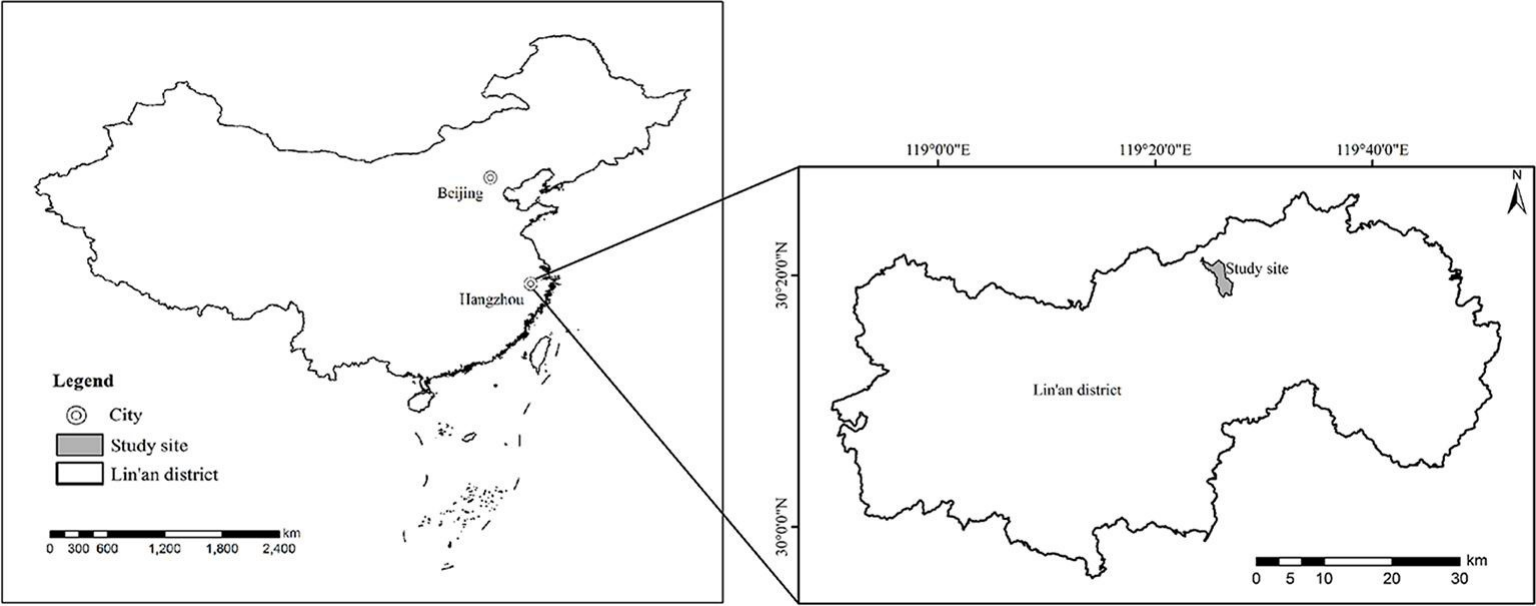
Location of the study area (Lin’an district, Hangzhou city, Zhejiang Province, China).

The study was conducted in an evergreen broad-leaved forest in the Tianmu Mountain National Nature Reserve, Lin’an district, Hangzhou city, Zhejiang Province, China. The experimental forest is located at latitudes from 30°18′30″ to 30°24′55″N, longitudes from 119°24′11″ to 119°28′21″E (Fig 1). The mean annual temperature at the experimental site ranges from 8.8°C to 14.8°C, mean annual rainfall ranges from 1390mm to 1870mm, mean annual solar radiation ranges from 3270 MJ∙m^-2^ to 4460 MJ∙m^-2^. Along a rising elevation gradient, the soil type transits from subtropical red soil to wet temperate brown-yellow soil, with red soil below 600m, yellow soil between 600m and 1200m, and brown-yellow soil above 1200m. The main forest types in the study region include evergreen broad-leaved forest, deciduous broad-leaved mixed forest, deciduous dwarf forest, coniferous broad-leaved mixed forest, and bamboo forest.

**Fig 2:**
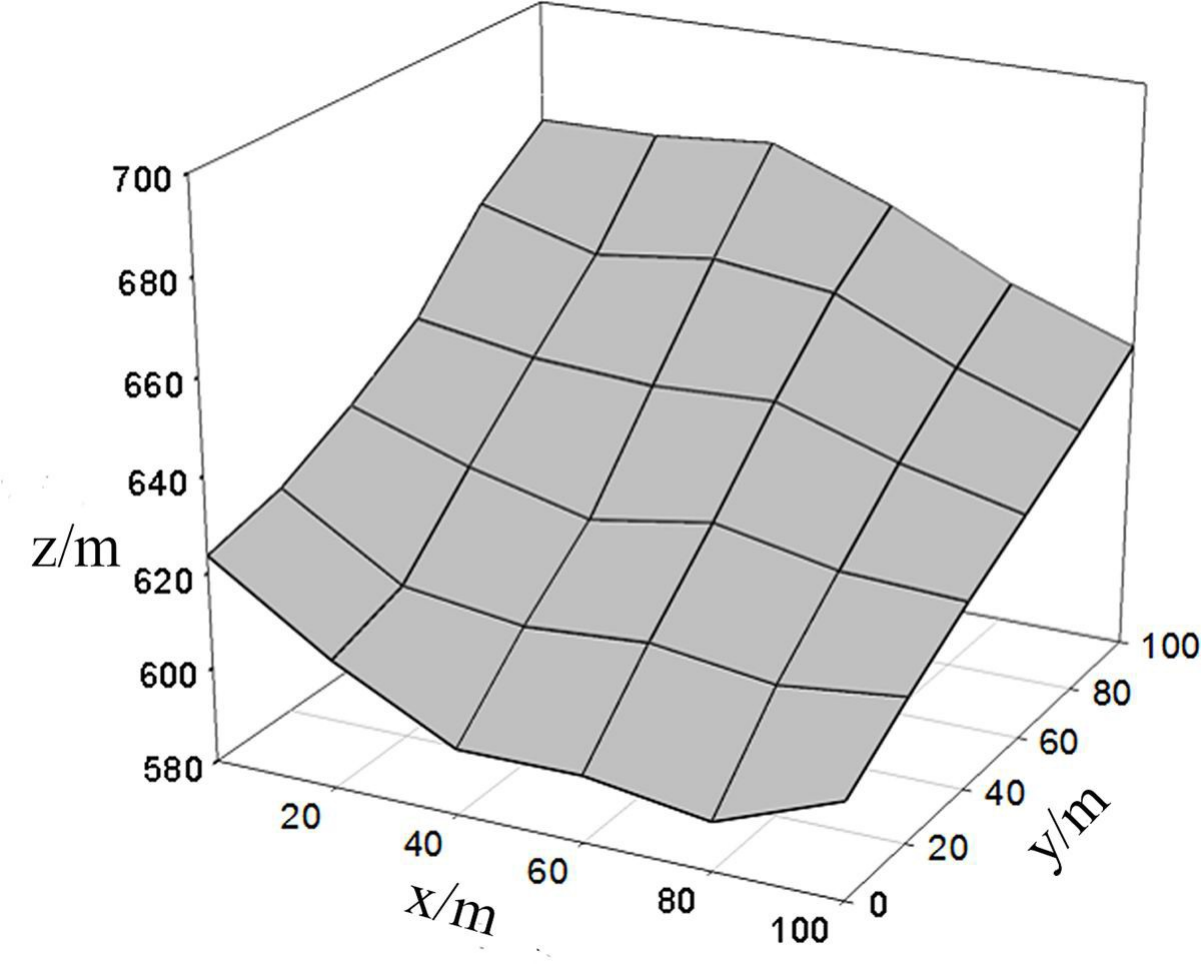
3D terrain map of the representative plot (100 m × 100 m) for the evergreen broad-leaved forest.

A permanent plot of 100m × 100m was established as a representative and set up from July to August in 2005. This plot was further divided into 25 subplots with an area of 20m × 20m using the adjacent grid survey method (Fig 2). Each subplot was used as an investigation unit to measure the properties of all trees in the plot. A full plot tree survey was conducted and the recorded items include species, coordinates (x, y, z), DBH, height and crown width of each tree.

### Vertical stratification

Zhou et al. [32] improved the criterion of forest strata stratification of the International Union of Forest Research Organizations, using the stand dominance height (h) as the stratification standard used. The average height of the highest 50 trees with DBH ≥ 5cm was used to represent the stand dominant height of the plot. Similarly, the minimum tree height (h_min_) was averaged based on the shortest 50 trees with DBH ≥ 5cm. Then, the height difference (Δh) between the stand dominant height and the minimum tree height was calculated. According to tree height (H), the trees with DBH ≥ 5cm were divided into the upper, middle and lower forest strata. Upper forest stratum is: H > h_min_+2/3Δh, middle forest stratum is: H ≥ h_min_+1/3Δh and ≤ h_min_+2/3Δh, lower forest stratum is: H < h_min_+1/3Δh. The plot inventory data indicated that the average stand dominant height was 17.2m, the minimum tree height was 1.5m and the difference value (Δh) was 15.7m. Therefore, the upper forest stratum is composed of trees with H > 12m, the middle forest stratum is composed of trees with H ≥ 6.7m and ≤ 12m, and the lower forest stratum is composed of trees with H < 6.7m.

### Stand structure indices

#### Non-spatial structure indices

The non-spatial structure index of each forest stratum was calculated based on the plot inventory data. Trees with DBH ≥ 5cm were defined as large trees, and DBH, tree height, crown width, density, and diversity were selected to represent the non-spatial structure.

The tree species diversity index is used to describe the proportion of species to individuals in a biological community. The tree species richness index was calculated according to Liu et al. [33]:

$$S=\frac{m-1}{\ln(M)},$$ (1)

where *S* is tree species richness index, *m* is the number of tree species in each subplot, and *M* is the total tree numbers in the plot.

#### Spatial structure indices

Mingling, competition index, and aggregation indices were selected to represent the characteristics of stand spatial structure, and a Voronoi diagram based on the relationship of neighborhood trees was used to calculate these stand spatial structure indices. In the Voronoi diagram, neighborhood trees are defined as the trees in the Thiessen polygons neighboring to the object Thiessen polygon [34]. The eight-neighborhood method is used to eliminate the edge effect [35].

The mingling index is used to represent the spatial isolation degree of tree species in a forest and is defined as the proportion of the number of trees that are not of the same species between nearest neighborhood tree species and object tree to the total neighborhood tree numbers [36]. The complete mingling (hereinafter referred to as mingling) was calculated according to Tang et al. [37]:

$$M_{i}=\frac{1}{n_{i}}\sum_{j=1}^{n_{i}}v_{ij};$$ (2)

$$Mc_{i}=\frac{1}{2}\left(D_{i}+\frac{c_{i}}{n_{i}}\right)\cdot M_{i},$$ (3)

where *M*_*i*_ is the mingling of the object tree *i*th; *M*_*ci*_ is the complete mingling of the object tree *i*th; *N*_*i*_ is the number of neighborhood trees; *V*_*ij*_ is a discrete variable, *V*_*ij*_ = 1 when the neighborhood tree *j*th and the object tree *i*th are different tree species, otherwise *V*_*ij*_ = 0; *c*_*i*_ is the number of pairs of nearest neighborhood trees that are not of the same species in the spatial structure unit where object tree *i*th is located; *D*_*i*_ is the Simpson diversity index of the tree species in the spatial structure unit where object tree *i*th is located.

The competition index is used to represent the competitive relationship among trees within a forest. The Hegyi competition index (hereinafter referred to as competition index) based on the Voronoi diagram was calculated according to Hegyi [38]:

$$CI_{i}=\sum_{j=1}^{n_{i}}\frac{d_{j}}{d_{i}\cdot L_{ij}};$$ (4)

$$CI=\frac{1}{Z}\sum_{i=1}^{Z}CI_{i},$$ (5)

where *CI*_*i*_ is the competition index of object tree *i*th, *L*_*ij*_ is the distance between object tree *i*th and neighborhood tree *j*th, *D*_*i*_ is the DBH of object tree *i*th, *D*_*j*_ is the DBH of neighborhood tree *j*th, *N*_*i*_ is the number of neighborhood trees in the spatial structure unit where object tree *i*th is located, *Z* is the number of object trees, *CI* is the stand competition index.

The aggregation index is used to represent the spatial distribution patterns in the forest and is defined as the proportion of the average distance between object trees and their nearest neighborhood trees to the expected average distance under a random tree distribution pattern [39]. It is calculated as:

$$R=\frac{\frac{1}{N}\sum_{i=1}^{N}r_{i}}{\frac{1}{2}\sqrt{\frac{F}{N}}},$$ (6)

where *R* is the aggregation index, *N* is the number of trees in the plot, *F* is the plot area, and *r*_*i*_ is the distance from the object tree *i*th to its nearest neighborhood tree.

#### Regeneration indicators

The trees with DBH < 5cm were defined as regenerated trees. According to the tree height and DBH, the regenerated trees were divided into three classes: seedlings, saplings, and young trees. Seedlings: H ≤ 1.5m and DBH < 1cm; Saplings: H ≤ 1.5m and DBH ≥ 1cm; young trees: H > 1.5m and DBH < 5cm [40]. The DBH or basal diameter, tree height, crown width, tree species richness index, and the number of regenerated trees were selected as regeneration indicators. The tree species richness index was also calculated using Eq (1).

### Data analysis

Redundancy analysis (RDA) is a direct gradient analysis method, which can intuitively investigate the complex relationship between multiple environmental factors and multiple species variables. The correlation between environmental factors and species variables is the product of the line length of species variables and the cosine of the angle between the environmental factors and species variables [33, 41]. The stand structure indices were taken as environmental variables, and regeneration indicators as species variables, the relationship between them were analyzed using the software Canoco 5 [42]. Firstly, to select a suitable model for RDA, the data were subjected to the detrended correspondence analysis. When the maximum gradient of the four axes was less than or equal to 3, the linear model was used; when the maximum gradient was equal to or greater than 4, the unimodal model was used; when the maximum gradient was between 3 and 4, both models could be selected [33, 43]. Secondly, log transformation and centralization were performed on the original data. Variance inflation factor was used to test the multicollinearity between variables, and the variance inflation factor was less than 20, which indicated that there was no multicollinearity among the stand structure indices. The most significant stand structure indices affecting regeneration were screened out through interactive forward selection. Finally, the specific relationship between the most significant stand structure indices and regeneration indicators was further analyzed using the “Multiple species response curves”.

## Results

### Structural characteristics of different forest strata

Stand structure characteristics of three forest strata were shown in Table 1. Most indices showed significant (p < 0.05) differences among three strata except for the aggregation index. The mingling index has significant differences between the upper and lower forest strata, and no significant differences between the middle and other forest strata. The tree species richness index has significant differences between the upper forest stratum and other forest strata, and no significant differences between the middle forest stratum and lower forest stratum. With the rising height of the forest strata, the mingling index increased, and the competition index and tree species richness index decreased. Therefore, it was reasonable to divide into three forest strata to study forest structure for this evergreen broad-leaved forest.

**Table 1:** Stand structure characteristics of different forest strata.

Stand	M	CI	R	N (trees.ha-1)	DBH (cm)	H (m)	W (m)	S
Upper	0.61±0.02a	4.13±0.50c	0.97±0.08a	139.00±19.10c	30.98±1.35a	14.80±0.41a	5.58±0.22a	0.86±0.14b
Middle	0.59±0.01ab	7.45±0.47b	0.92±0.04a	548.00±44.25b	15.83±0.63b	8.50±0.09b	4.18±0.21b	2.41±0.15a
Lower	0.57±0.01b	10.49±0.37a	0.97±0.03a	942.00±75.96a	8.20±0.23c	5.02±0.04c	2.92±0.09c	2.44±0.15a
Whole	0.58±0.01	8.93±0.26	0.95±0.02	1629.00±102.38	12.70±0.34	7.17±0.17	3.58±0.15	3.72±0.20

Different letters in the same column indicate a significant difference at p < 0.05 level. M: mingling index; CI: competition index; R: aggregation index; N: density; DBH: diameter at breast height; H: tree height; W: crown width; S: tree species richness index.

### Effects of structure on regeneration at a whole stand level

The results of RDA showed that 49.22% of the regeneration variation was explained by the whole stand structure with 25.23% being explained by the first axis and 19.68% by the second axis, indicating that the correlation between whole stand structure indices and regeneration was mainly determined by the first and second axis (Table 2). DBH, tree species richness index, and crown width had the most important effect on regeneration, with the contributions of 18.4%, 9.4%, and 7.2%, respectively. The total contributions of these three indices accounted for 71.11% of the explained variation.

**Table 2:** Summary of redundancy analysis results for the contributions of different structure indices to regeneration at the whole stand level.

Name	Mean	Stand. dev.	Inflation factor	Explains %	Contribution %	F	P
Wh_D	12.69	1.68	3.78	18.4	36.1	5.2	0.006***
Wh_S	3.58	0.72	6.34	9.4	18.4	2.9	0.048**
Wh_W	3.72	0.96	3.97	7.2	14.1	2.3	0.07*
Wh_CI	8.63	2.16	2.30	5.7	11.1	1.9	0.126
Wh_R	1.00	0.22	3.21	3.9	7.6	1.3	0.256
Wh_H	7.11	0.82	5.21	2.6	5.1	0.8	0.422
Wh_M	0.57	0.07	2.10	2.4	4.8	0.8	0.484
Wh_N	1629.00	501.58	4.14	1.4	2.8	0.5	0.762
				Axis 1	Axis 2	Axis 3	Axis 4
Eigenvalues				0.2523	0.1968	0.0276	0.0156
Explained variation (cumulative)				25.23	44.91	47.66	49.22

Wh_M, Wh_CI, Wh_R, Wh_S, Wh_DBH, Wh_H, Wh_W and Wh_N denote the mingling index, competition index, aggregation index, tree species richness index, diameter at breast height, tree height, crown width and density, respectively.

***: p < 0.01

**: p < 0.05

*: p < 0.1.

**Fig 3:**
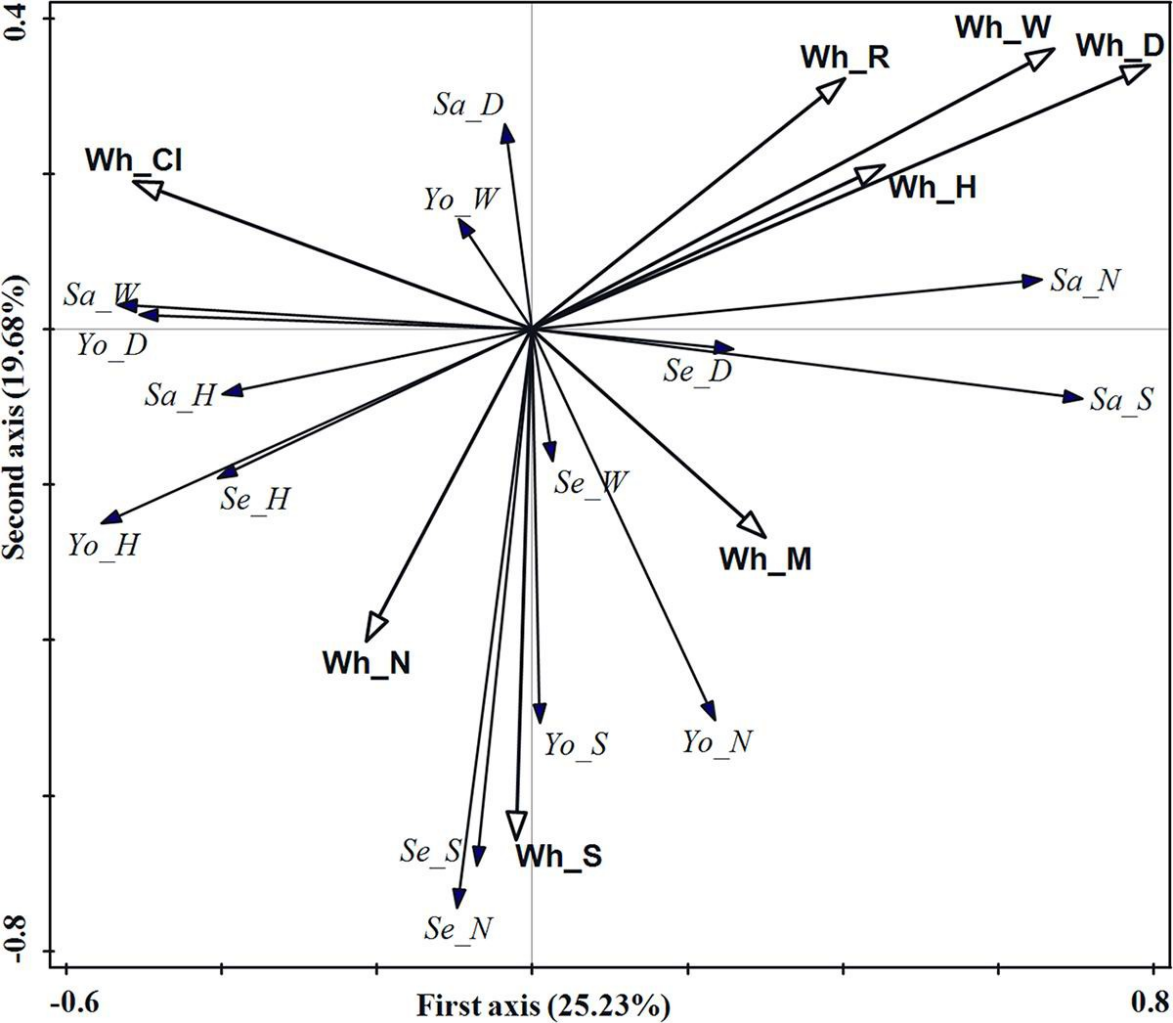
RDA ordination diagram of whole stand structure indices and regeneration in the evergreen broad-leaved forest. Hollow arrows represent stand structure indices and solid arrows represent regeneration indicators. Wh_M, Wh_CI, Wh_R, Wh_S, Wh_DBH, Wh_H, Wh_W and Wh_N denote the mingling index, competition index, aggregation index, tree species richness index, diameter at breast height, tree height, crown width and density, respectively. Se_N, Se_D, Se_H, Se_W and Se_S denote seedling density, basal diameter, tree height crown width and tree species richness index, respectively; Sa_N, Sa_D, Sa_H, Sa_W and Sa_S denote sapling density, basal diameter, tree height, crown width and tree species richness index, respectively; Yo_N, Yo_D, Yo_H, Yo_W and Yo_S denote young tree density, diameter at breast height, tree height, crown width and tree species richness index, respectively.

The ordination diagram of RDA showed that the DBH and crown width of the whole stand had a strong positive effect on sapling density and tree species richness index, and a strong negative effect on young tree height and DBH (Fig 3). The tree species richness index of the whole stand had a strong positive effect on the density and tree species richness index of both seedlings and young trees.

**Fig 4:**
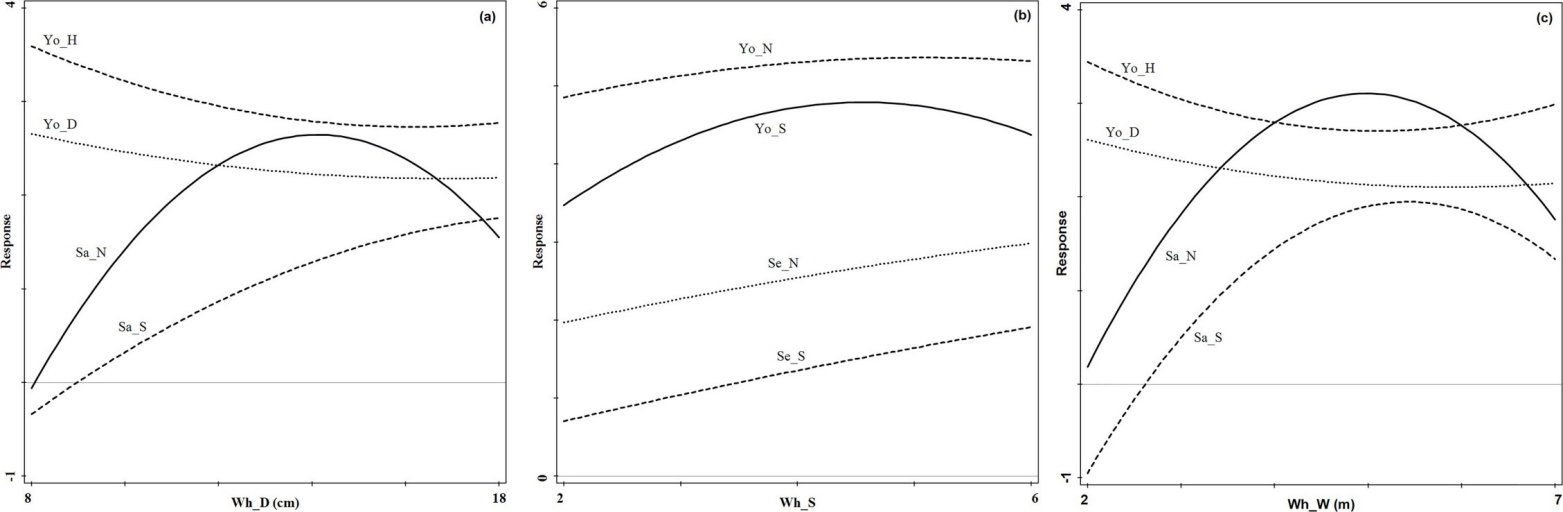
Regeneration response curves to the identified most important structure indices at the whole stand level (Fig 4A: Wh_D; Fig 4B: Wh_S; Fig 4C: Wh_W). Se_N and Se_S denote seedling density and tree species richness index, respectively; Sa_N and Sa_S denote sapling density and tree species richness index, respectively; Yo_N, Yo_D, Yo_H and Yo_S denote young tree density, diameter at breast height, tree height and tree species richness index, respectively.

The specific effects of the dominant whole stand structure indices on regeneration were shown in Fig 4. With the changing DBH, young tree height and DBH showed a decreasing response curve, sapling tree species richness index showed an increasing trend, and sapling density showed a unimodal distribution. Sapling density maintained a high response value when the DBH of the whole stand was between 13cm and 15cm, suggesting that the DBH ranging 13–15 cm was more favourable for the regeneration of saplings (Fig 4A). With the increase of the tree species richness index in the whole stand, seedling density and tree species richness index showed an increasing trend, and young tree density and tree species richness index showed a unimodal distribution. The young tree density and species richness index kept a high response value when the tree species richness index of the whole stand was between 4 and 5 (Fig 4B). With the increase of the crown width in the whole stand, young tree DBH and height showed a single trough distribution pattern, and the minimum response to the crown width of the whole stand was located at values between 4m and 5.5m; while sapling density and tree species richness index showed a unimodal distribution pattern, and the maximum response to the crown width of the whole stand was located at values between 4.5m and 6m (Fig 4C).

### Effects of structure on regeneration at a forest stratum level

#### Effects of upper forest stratum structure

The results of RDA showed that 37.76% of the regeneration variation can be explained by the upper forest stratum structure index with 19.83% being explained by the first axis and 10.87% being explained by the second axis. RDA can better explain the relationship between the upper forest stratum structure index and regeneration. The interactive forward selection results of the upper forest stratum showed that the tree height was the most significant structure factor affecting regeneration, and the contribution was 13.9%, which accounts for about 36.81% of total interpretive ability of the all upper forest stratum structure indices (Table 3).

**Table 3:** Summary of redundancy analysis results for the contributions of different upper stratum structure indices to regeneration in the evergreen broad-leaved forest.

Name	Mean	Stand. dev.	Inflation factor	Explains %	Contribution %	F	P
Up_H	14.8	2.0	2.2	13.9	35.9	3.7	0.02**
Up_N	139.0	93.6	9.7	6.5	16.7	1.8	0.134
Up_M	0.6	0.1	1.8	6.3	16.3	1.8	0.116
Up_CI	3.9	2.7	2.0	4.5	11.5	1.2	0.288
Up_W	5.6	1.1	2.0	2.8	7.2	0.8	0.480
Up_R	0.9	0.5	2.2	2.3	5.8	0.6	0.628
Up_S	0.9	0.7	10.7	1.6	4.2	0.5	0.772
Up_D	30.98	6.6	2.3	0.9	2.3	0.2	0.946
			Axis 1	Axis 2	Axis 3	Axis 4	
Eigenvalues			0.1983	0.1087	0.0578	0.0128	
Explained variation (cumulative)			19.83	30.7	36.48	37.76	

Up_M, Up_CI, Up_R, Up_S, Up_DBH, Up_H, Up_W and Up_N denote the mingling, competition index, aggregation index, tree species richness index, diameter at breast height, tree height, crown width and density, respectively.

***: p < 0.01

**: p < 0.05

*: p < 0.

**Fig 5:**
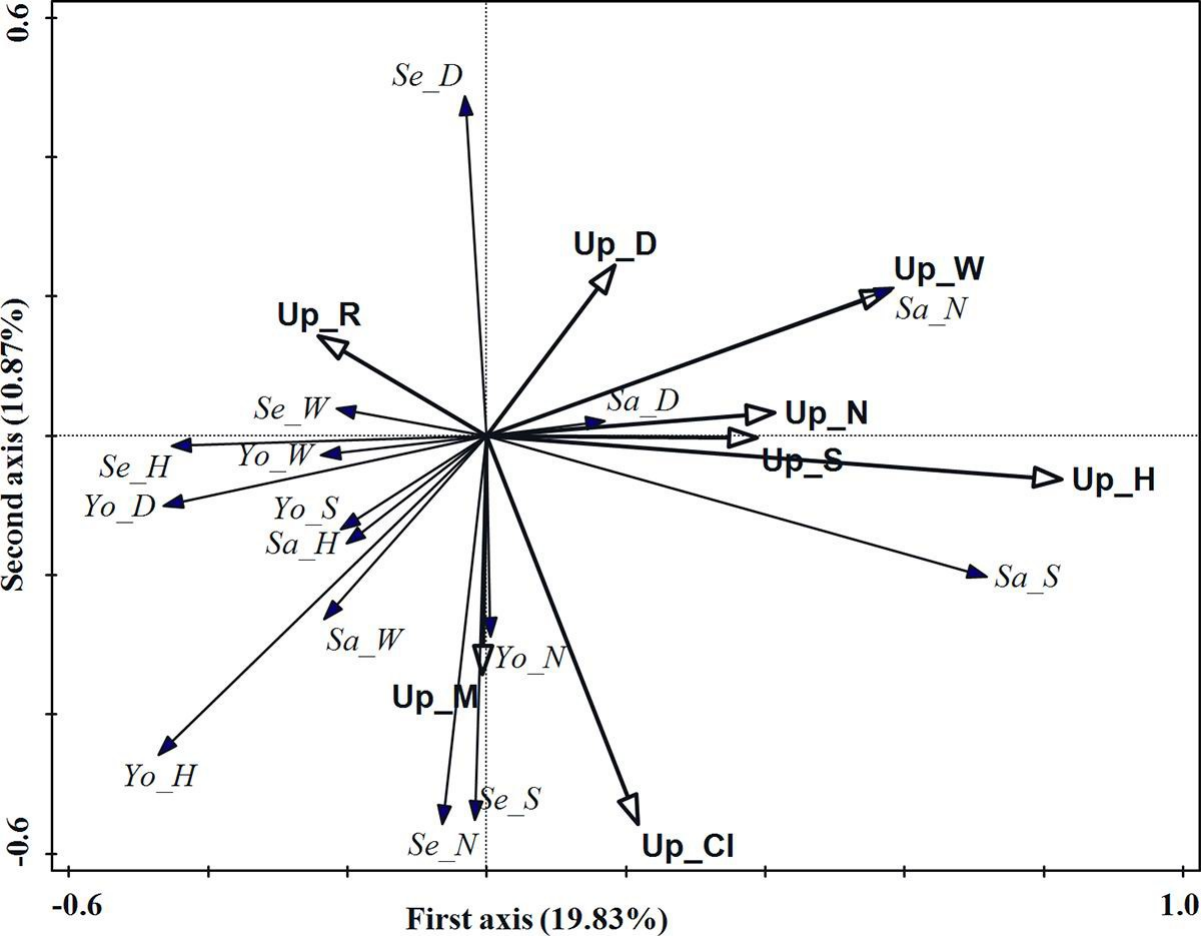
RDA ordination diagram of upper forest stratum structure indices and regeneration in the evergreen broad-leaved forest. Hollow arrows represent stand structure indices and solid arrows represent regeneration indicators. Up_M, Up_CI, Up_R, Up_S, Up_DBH, Up_H, Up_W and Up_N denote the mingling, competition index, aggregation index, tree species richness index, diameter at breast height, tree height, crown width and density, respectively. Se_N, Se_D, Se_H, Se_W and Se_S denote seedling density, basal diameter, tree height crown width and tree species richness index, respectively; Sa_N, Sa_D, Sa_H, Sa_W and Sa_S denote sapling density, basal diameter, tree height, crown width and tree species richness index, respectively; Yo_N, Yo_D, Yo_H, Yo_W and Yo_S denote young tree density, diameter at breast height, tree height, crown width and tree species richness index, respectively.

**Fig 6:**
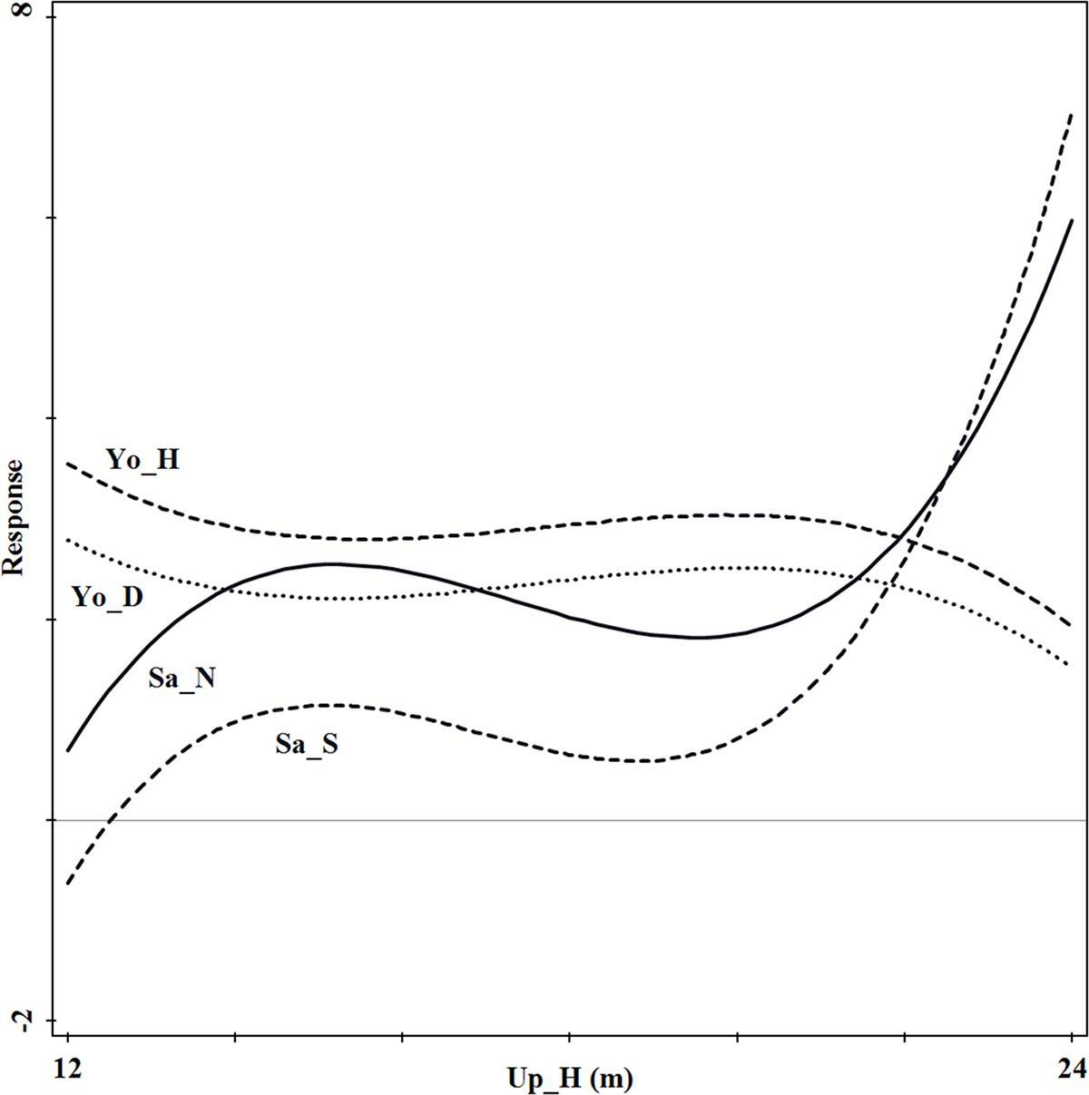
Regeneration response curves to the most important upper forest stratum structure index (Up_H). Sa_N and Sa_S denote sapling density and tree species richness index, respectively; Yo_D and Yo_H denote young tree diameter at breast height and tree height, respectively.

According to the ordination diagram of RDA (Fig 5), the tree height of the upper forest stratum had a greater positive effect on sapling density and tree species richness index, and a greater negative effect on young tree height and DBH. When the tree height ranged between 12m and 16.5m, the young tree height and DBH had a single valley distribution, while sapling density and tree species richness index had a unimodal distribution. When the tree height ranged between 16.5m and 24m, young tree height and DBH had a unimodal distribution, while sapling density and tree species richness index had a single valley distribution (Fig 6).

#### Effect of middle forest stratum structure

The redundancy analysis results of middle forest stratum structure and regeneration were shown in Table 4. 41.45% of the regeneration variation can be explained by the four axes, 38.26% of the regeneration variation can be explained by the first two axes with 23.12% being explained by the first axis and 15.14% being explained by the second axis. The most important structure factors were screened out by the interactive forward selection as follows: the tree species richness index and crown width, which explained 16.7% and 14.5% of the regeneration variation, respectively. These two factors accounted for 75.27% of the total explained variation of all middle forest stratum structure indices.

**Table 4:** Summary of redundancy analysis results for the contributions of different middle stratum indices to regeneration in the evergreen broad-leaved forest.

Name	Mean	Stand. dev.	Inflation factor	Explains %	Contribution %	F	P
Mid_S	2.4	0.7	3.8	16.7	39.0	4.6	0.006***
Mid_W	4.2	1.0	1.7	14.5	33.8	4.6	0.012**
Mid_D	15.8	3.1	1.8	4.2	9.7	1.4	0.262
Mid_M	0.6	0.1	1.9	2.5	5.7	0.8	0.434
Mid_CI	6.8	2.8	3.1	1.6	3.8	0.5	0.636
Mid_H	8.5	0.4	1.9	1.6	3.7	0.5	0.694
Mid_N	548.0	216.8	3.6	1.0	2.3	0.3	0.886
Mid_R	0.98	0.3	2.9	0.8	2.0	0.2	0.934
			Axis 1	Axis 2	Axis 3	Axis 4	
Eigenvalues			0.2312	0.1514	0.0182	0.0137	
Explained variation (cumulative)			23.12	38.26	40.08	41.45	

Mid_M, Mid_CI, Mid_R, Mid_S, Mid_DBH, Mid_H, Mid_W and Mid_N denote the mingling, competition index, aggregation index, tree species richness index, diameter at breast height, tree height, crown width and density, respectively.

***: p < 0.01

**: p < 0.05; *: p < 0.1.

**Fig 7:**
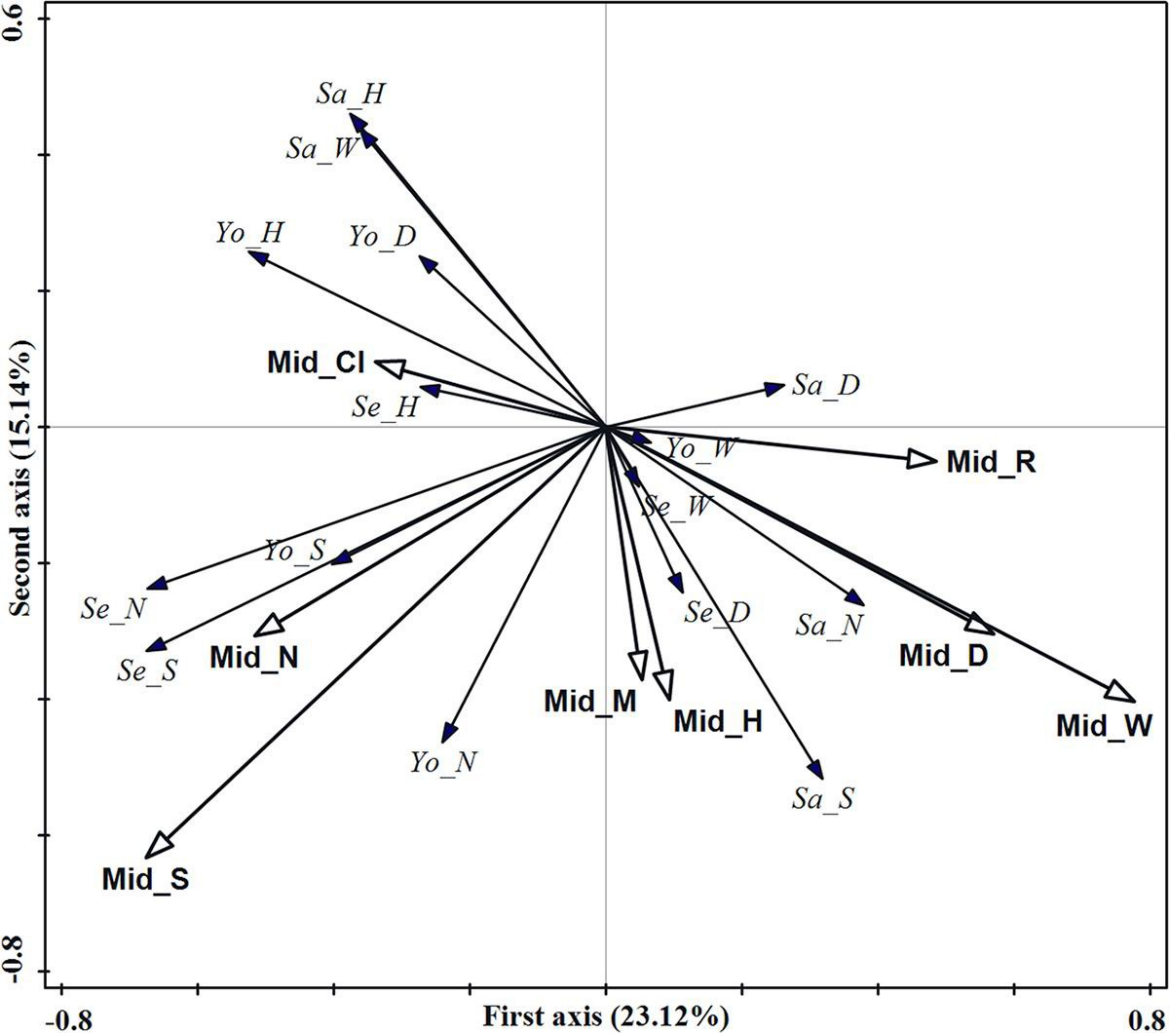
RDA ordination diagram of middle forest stratum structure indices and regeneration in the evergreen broad-leaved forest. Hollow arrows represent stand structure indices and solid arrows represent regeneration indicators. Mid_M, Mid_CI, Mid_R, Mid_S, Mid_DBH, Mid_H, Mid_W and Mid_N denote the mingling, competition index, aggregation index, tree species richness index, diameter at breast height, tree height, crown width and density, respectively. Se_N, Se_D, Se_H, Se_W and Se_S denote seedling density, basal diameter, tree height, crown width and tree species richness index, respectively; Sa_N, Sa_D, Sa_H, Sa_W and Sa_S denote sapling density, basal diameter, tree height, crown width and tree species richness index, respectively; Yo_N, Yo_D, Yo_H, Yo_W and Yo_S denote young tree density, diameter at breast height, tree height, crown width and tree species richness index, respectively.

The tree species richness index of the middle forest stratum had a larger positive effect on both seedling and young tree species richness index and density (Fig 7). The crown width had a larger positive effect on sapling tree species richness index and density and had a larger negative effect on young tree height, and sapling crown width and height.

**Fig 8:**
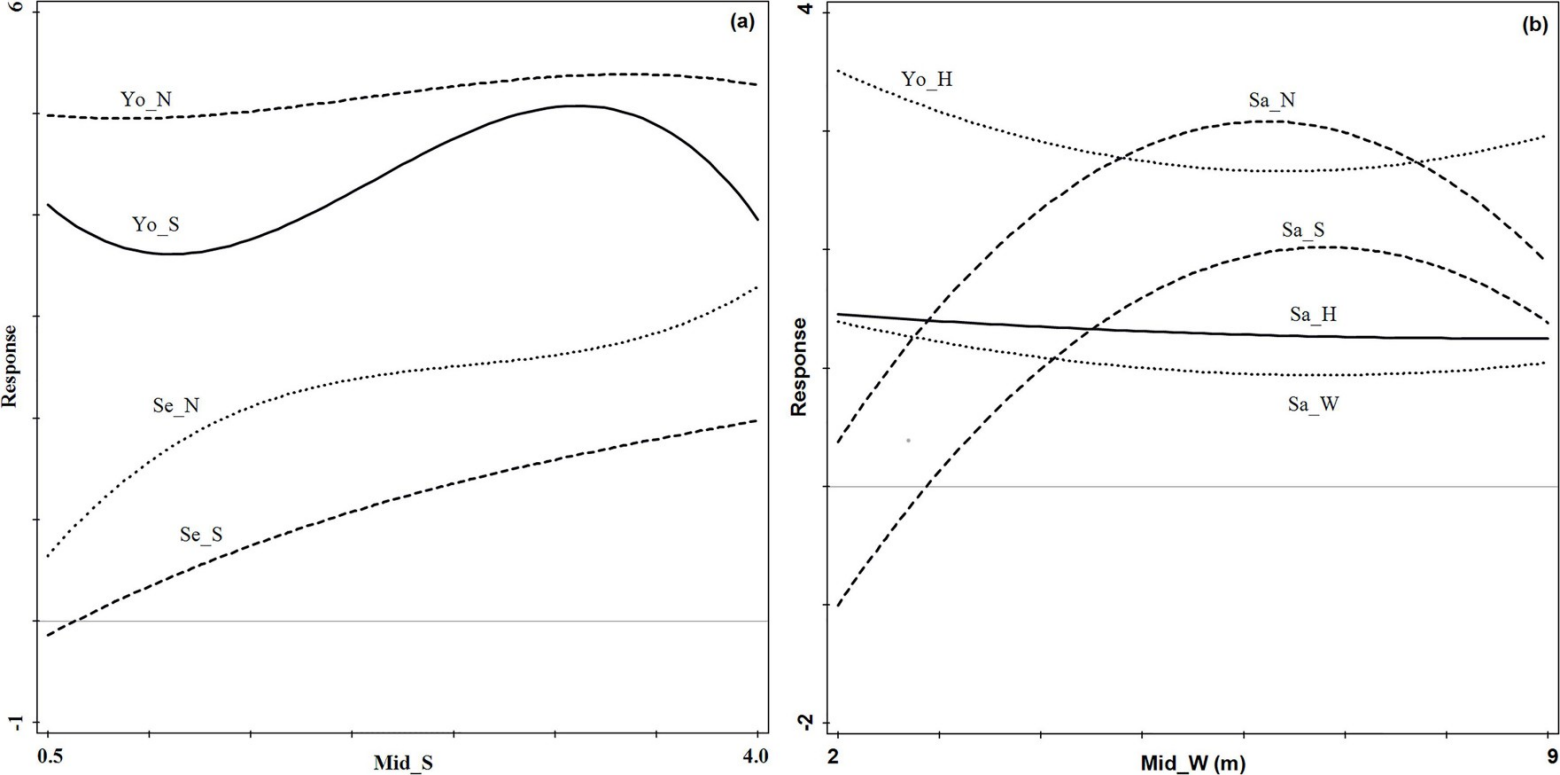
Regeneration response curves to the most important middle forest stratum structure indices in the evergreen broad-leaved forest (Fig 8A: Mid_S; Fig 8B: Mid_W). Se_N and Se_S denote seedling density and tree species richness index, respectively; Sa_N, Sa_H, Sa_W and Sa_S denote sapling density, tree height, crown width and tree species richness index, respectively; Yo_N, Yo_H and Yo_S denote young tree density, tree height and tree species richness index, respectively.

The young tree density, seedling density and species richness index did not change much with increasing tree species richness index of the middle forest stratum (Fig 8). The young tree species richness index reached the minimum value when the species richness index ranged between 1 and 1.5 and reached the maximum value when the tree species richness index ranged between 3 and 3.5 (Fig 8A). With the increase of crown width, the sapling height showed a slightly declining trend, the sapling crown width and young tree height showed a single valley distribution, and the sapling density and tree species richness index showed a unimodal distribution. When the crown width ranged between 6m to 7m, the young tree height and sapling crown width reached the minimum value, and the sapling density and species richness diversity reached the maximum value (Fig 8B).

#### Effect of lower forest stratum structure

The redundancy analysis results of lower forest stratum structure and regeneration were shown in Table 5. 49.58% of the regeneration variation can be explained by the four axes, 43.78% of the regeneration variation can be explained by the first two axes with 30.09% being explained by the first axis and 13.69% by the second axis. Therefore, the first two axes provided an optimal explanation for the variation in both lower forest stratum structure indices and regeneration. From the forward selection results of the lower forest stratum, the most significant structure factors affecting regeneration were: crown width, competition index, tree height and tree species richness index, with explanation rates of 11.2%, 10.8%, 9.5%, and 7.2%, respectively. These factors accounted for 78.06% of the total explained variation of all lower forest stratum structure indices.

**Table 5:** Summary of redundancy analysis results for the contributions of different lower stratum indices to regeneration at the lower stratum in the evergreen broad-leaved forest.

Name	Mean	Stand. dev.	Inflation factor	Explains %	Contribution %	F	P
Low_W	2.9	0.4	1.6	11.2	21.5	2.9	0.040**
Low_CI	10.3	3.3	1.6	10.8	20.9	3.3	0.034**
Low_H	5.0	0.2	1.3	9.5	18.3	2.6	0.044**
Low_S	2.4	0.8	3.6	7.2	13.8	2.3	0.070*
Low_M	0.6	0.1	2.1	3.9	7.4	1.3	0.280
Low_N	942.0	372.1	3.7	3.9	7.6	1.3	0.252
Low_D	8.2	1.1	1.3	3.8	7.3	1.3	0.236
Low_R	1.0	0.3	2.1	1.6	3.1	0.5	0.688
			Axis 1	Axis 2	Axis 3	Axis 4	
Eigenvalues			0.3009	0.1369	0.0423	0.0157	
Explained variation (cumulative)			30.09	43.78	48.01	49.58	

Low_M, Low_CI, Low_R, Low_S, Low_DBH, Low_H, Low_W and Low_N denote the mingling, competition index, aggregation index, tree species richness index, diameter at breast height, tree height, crown width and density, respectively. ***: p < 0.01

**: p < 0.05

*: p < 0.1.

**Fig 9:**
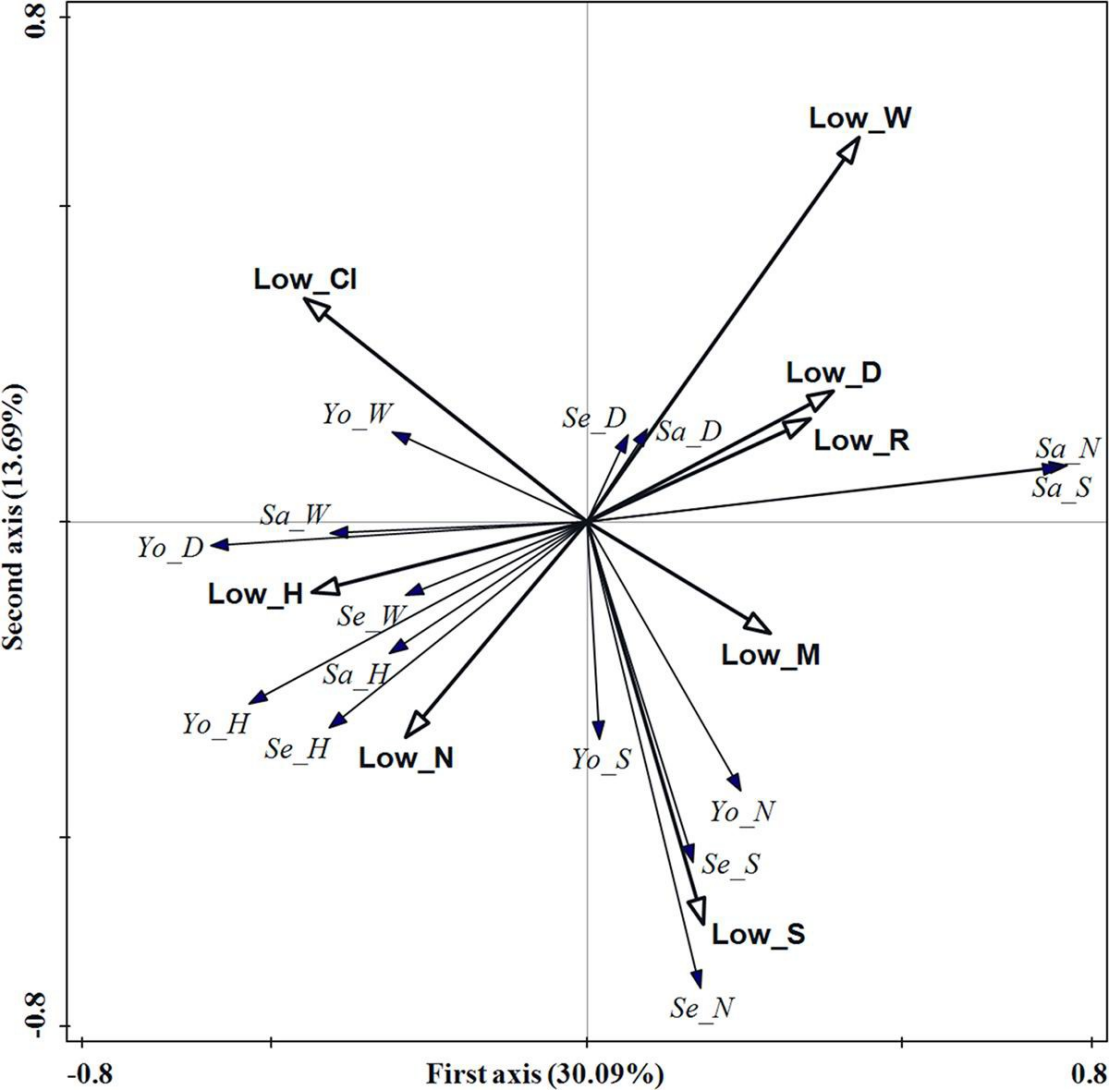
RDA ordination diagram of lower forest stratum structure indices and regeneration in the evergreen broad-leaved forest. Hollow arrows represent stand structure indices and solid arrows represent regeneration indicators. Low_M, Low_CI, Low_R, Low_S, Low_DBH, Low_H, Low_W and Low_N denote the mingling, competition index, aggregation index, tree species richness index, diameter at breast height, tree height, crown width and density, respectively. Se_N, Se_D, Se_H, Se_W and Se_S denote seedling density, basal diameter, tree height, crown width and tree species richness index, respectively; Sa_N, Sa_D, Sa_H, Sa_W and Sa_S denote sapling density, basal diameter, tree height, crown width and tree species richness index, respectively; Yo_N, Yo_D, Yo_H, Yo_W and Yo_S denote young tree density, diameter at breast height, tree height, crown width and tree species richness index, respectively.

The crown width of the lower forest stratum had a greater positive effect on sapling density and tree species richness index and a greater negative effect on seeding density and tree species richness index, and young tree density and height (Fig 9). The competition index had a greater negative effect on regeneration of tree density and tree species richness index. The tree height had a greater negative effect on sapling density and tree species richness index, and a positive effect on young tree height and DBH. The tree species richness index had a greater positive effect on seeding and young tree density and species richness index.

**Fig 10:**
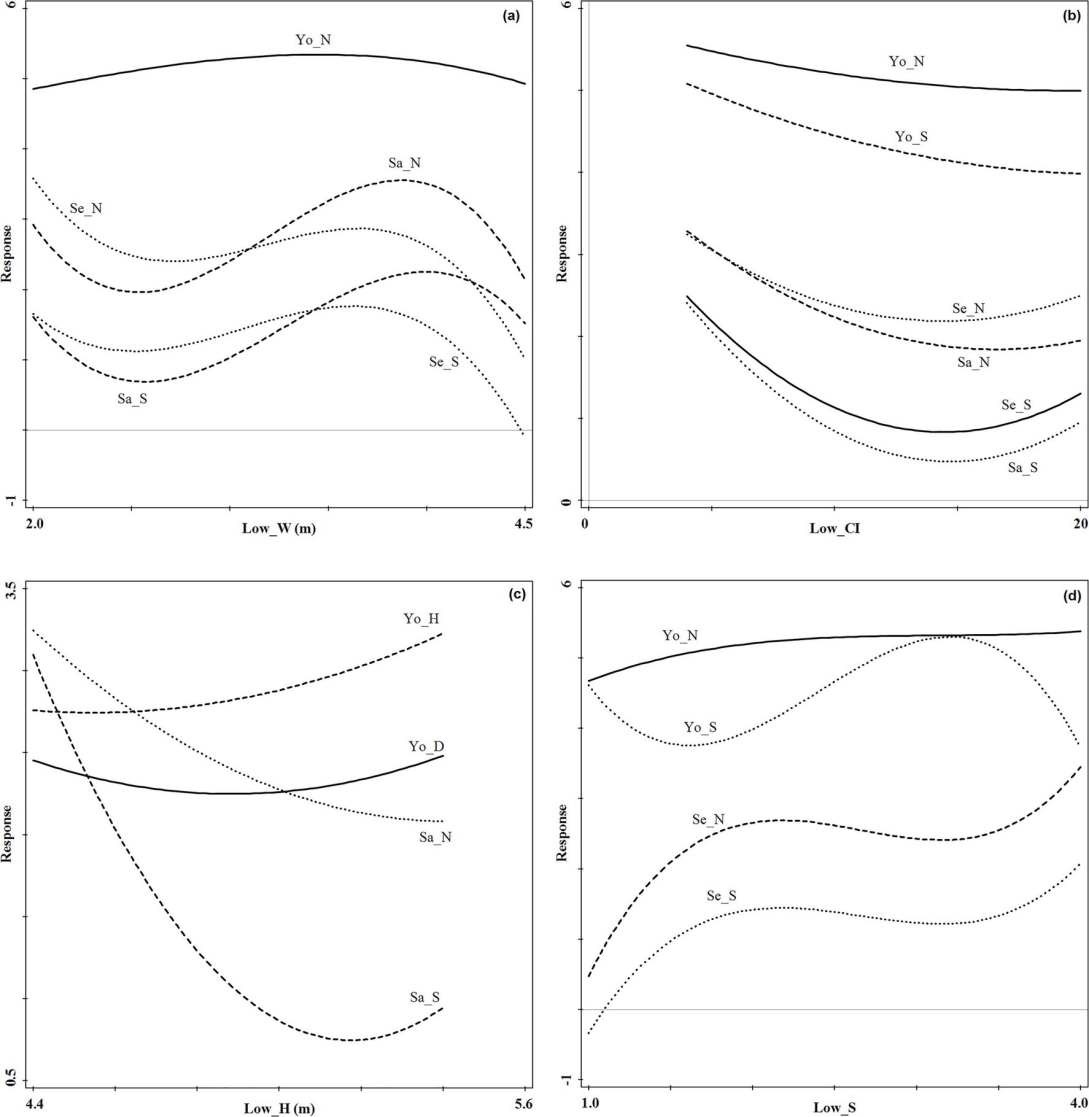
Regeneration response curves to the most important lower forest stratum structure indices in the evergreen broad-leaved forest (Fig 10A: Low_W; Fig 10B: Low_Cl; Fig 10C: Low_H; Fig 10D: Low_S). Se_N and Se_S denote seedling density and tree species richness index, respectively; Sa_N and Sa_S denote sapling density and tree species richness index, respectively; Yo_N, Yo_D, Yo_H and Yo_S denote young tree density, diameter at breast height, tree height and tree species richness index, respectively.

Fig 10 showed the specific effects of the most important structure indices on regeneration at the lower forest stratum. When the crown width of the lower forest stratum ranged between 2.0m and 3.2m, the seeding density and tree species richness index, sapling density, and tree species richness index had a single valley distribution, sapling density, and tree species richness index reached the minimum value. When the crown width ranged between 3.2m and 4.5m, the seeding density and tree species richness index, and sapling density and tree species richness index had a unimodal distribution, but only sapling density and tree species richness index maintained a high response value (Fig 10A). With the increase of competition index, the seedling and sapling density and tree species richness index had a single valley distribution, and the young tree density and tree species richness index showed a decreasing trend. The seedling and sapling density and tree species richness index reached the minimum value when the competition index ranged between 13 and 16 (Fig 10B). With the increase of tree height, the sapling density showed a decreasing trend, young tree DBH and sapling tree species richness index showed a single valley distribution, and young tree height showed an increasing trend. The sapling tree species richness index reached the minimum value when the tree height ranged between 5m and 5.3m. The young tree DBH reached the minimum value when the tree height ranged between 4.8m and 5m (Fig 10C). When the tree species richness index ranged between 1.0 and 2.5, the young tree density showed an increasing trend, the seedling density and tree species richness index showed a unimodal distribution, and the young tree species richness index showed a single valley distribution. When the tree species richness index was between 2.5 and 4.0, the young tree density kept relatively stable, the seedling density and tree species richness index showed a single valley distribution, and the young tree species richness index showed a unimodal distribution and reached the maximum value (Fig 10D).

## Discussion

Investigation and analysis of stand structure can help us understand the history, current situation, and future development of forest ecosystems and thus carry out appropriate management practices [26, 30]. The structural characteristics of the stand affect the spatial and temporal distribution of regeneration, the quantity and quality of provenance, and the growth and death of individuals [4, 6, 44]. Many researchers have concluded that stand density had both positive and negative effects on regeneration [10, 12] and species composition directly affected the abundance and composition of regeneration [7, 45]. Vertical and horizontal differences resulting from spatial distribution, mixture, and competition determine spatial variation in microclimate and structural complexity, and thus directly and indirectly affect the survival and abundance of plant species [46]. The structure management on forests aims to improve forest productivity and quality by optimizing forest spatial structure parameters, thus achieving sustainable utilization [47]. As inferred from our study, the combinations of the spatial and non-spatial structure to explore the impact of different forest strata structure on regeneration can deepen our understanding of the specific relationships between forest strata and regeneration, and thus provide scientific guides for sustainable forest management.

### Effect of whole stand structure on regeneration

In the evergreen broad-leaved forest in Tianmu Mountain, DBH, tree species richness index and crown width were the main indices affecting regeneration. The DBH and crown width could inhibit the individual size growth of regenerated trees, but to a certain extent could also promote the regeneration of young tree density and species richness index, which is consistent with many previous studies [14, 21, 48]. It is generally believed that the larger DBH and crown width, the older stand age and the more mature seed trees in the forest can provide more favorable resources for regeneration. Some previous studies showed that crown width plays a role of shading and shelter for regeneration and affects the growth of regenerated trees by changing habitat conditions such as light and humidity in the forest [49–51]. Our results clearly showed that both the larger and smaller crown width could inhibit the regeneration of seedling density, sapling density and sapling tree species richness index, but could promote the growth of young tree height and DBH. Small crown width causes abundant sunlight to reach the forest floor directly, and some regenerated trees lose moisture easily. Under this condition, some intolerant tree species may compete strongly with regenerated trees for the available resources, thereby reducing survival or growth rates of regenerated trees [44]. If the crown width is large, the photosynthesis of the regenerated trees is reduced due to a lack of adequate sunlight. Tree species richness was one of the main drivers affecting regeneration [7, 52]. In this study, the tree species richness index of the whole stand was positively correlated with the density and species richness of the regenerated trees. This can be explained that different tree species have different ways of regeneration [53], and the seed size and quality also have certain differences [54], making them adaptable to different habitats. Therefore, in the management of this evergreen broad-leaved forest regeneration in the future, the DBH, crown width and tree species richness index of the whole stand can be reasonably regulated according to the needs of the management objectives to promote the regenerated trees at different stages of growth.

### Effect of forest strata structure on regeneration

The vertical stratification of the canopy is a forest attribute that influences both tree growth and understory community structure [16, 17]. Different forest strata have their own functions and roles. In the upper forest stratum, the tree height was the main stand structure factor affecting regeneration. In the middle forest stratum, the tree species richness index and crown width were the main stand structure indices affecting regeneration. In the lower forest stratum, the crown width, competition index, tree height, and tree species richness index were the main stand structure indices affecting regeneration. The shade-casting ability of the overstory and the competitiveness of the understory were more important than the abundance of these layers *per se* in determining the process of tree regeneration, especially the possibility of presence [44]. In terms of the response curves of the regenerated trees at different stages, seedlings and saplings had more obvious fluctuations in the structure indicators. Compared to the effects of forest strata and the whole stand on regeneration, it is observed that the tree species richness index and crown width of the whole stand play a sheltered role for regeneration trees and provide the seed source of dominant tree species which mainly comes from the middle and lower forest strata. Because the main dominant tree species (*C*. *gracilis*, *C*. *glauca*, *L*. *brevicaudatus*, *and C*. *fraterna*) in the middle and the lower forest strata had more tree numbers and stronger natural regeneration ability, while tree numbers in the upper forest stratum were relatively smaller. In addition to some of the main dominant species in the upper forest stratum, there are also a few other dominant species, such as *Quercus fabri*, *Liquidambar formosana*, *Cunninghamia lanceolata*, and *Torreya grandis*. The openness of object trees represents the light intensity in the forest where the object tree is located, and is defined as the sum of the proportion of the distance between the object tree and its neighborhood trees to the neighborhood tree height [29, 55]. This indicates that the light intensity of a certain site in the forests is largely determined by the neighborhood trees height and the distance between the neighborhood trees and object trees. The tree height of the upper and lower forest strata had a significant impact on regeneration, because the tree height of the upper forest stratum is too high, which may cause a decrease of the light intensity and temperature in the forest, inhibiting the individual size growth of regenerated trees. The increase of tree height of the lower forest stratum can provide more growth space for regenerated trees and reduce the competition for available resources, thus promote the individual size growth of regenerated trees.

We found that the regeneration of evergreen broad-leaved forest in Tianmu Mountain was mainly affected by non-spatial structure factors, while the spatial structure factors only had a significant impact on the regeneration through the competition index of the lower forest stratum. There was no significant difference in the spatial distribution pattern of different forest strata, which may be the reason why the spatial distribution pattern of the evergreen broad-leaved forest had no effect on regeneration. This research found that the smaller the competition index, the higher regenerated tree species diversity and density. The reason is that air temperature and humidity were mainly influenced by understory layers [25]. Meanwhile, the niche of trees in the lower forest stratum overlapped with that of regeneration trees, leading to the intensive competition for nutrients, living space and other resources among regenerated trees, which resulted in a significant effect of the competition index of the lower forest stratum on regeneration.

## Conclusions

The results of this study showed that with the decrease of forest strata, the number of significant structural factors affecting regeneration increased. Different regeneration indicators had different responses to the main stand structure indices, while the young tree height and DBH, and the tree species diversity and density of regeneration trees were most affected by the main stand structure indices, but seedling and sapling were more sensitive to the change of the same structure factor than young tree. The order of forest stratum structure effect on regeneration was: lower forest stratum > middle forest stratum > upper forest stratum. Hence different management practices should be formulated for different forest strata to effectively improve the regeneration ability and thus restore the evergreen broad-leaved forest in Tianmu Mountain.
